# Sodium-Glucose Co-Transporter 2 Inhibitors in Heart Failure—Current Evidence in Special Populations

**DOI:** 10.3390/life13061256

**Published:** 2023-05-25

**Authors:** Gassan Moady, Tuvia Ben Gal, Shaul Atar

**Affiliations:** 1 Department of Cardiology, Galilee Medical Center, Nahariya 2210001, Israel; shaula@gmc.gov.il; 2 Azrieli Faculty of Medicine, Bar Ilan University, Safed 5290002, Israel; 3 Heart Failure Unit, Cardiology Department, Rabin Medical Center, Petah Tikva 4941492, Israel; bengaltu@gmail.com; 4 Sackler Faculty of Medicine, Tel Aviv University, Tel Aviv 6997801, Israel

**Keywords:** heart failure, sodium-glucose co-transporter, diabetes, cardiovascular outcomes

## Abstract

Sodium-glucose co-transporter 2 (SGLT2) inhibitors, originally used for diabetes mellitus, are gaining more popularity for other indications, owing to their positive cardiovascular and renal effects. SGLT2 inhibitors reduce heart failure (HF) hospitalization and improve cardiovascular outcomes in patients with type 2 diabetes. Later, SGLT2 inhibitors were evaluated in patients with HF with reduced ejection fraction (HFREF) and had beneficial effects independent of the presence of diabetes. Recently, reductions in cardiovascular outcomes were also observed in patients with HF with preserved ejection fraction (HFPEF). SGLT2 inhibitors also reduced renal outcomes in patients with chronic kidney disease. Overall, these drugs have an excellent safety profile with a negligible risk of genitourinary tract infections and ketoacidosis. In this review, we discuss the current data on SGLT2 inhibitors in special populations, including patients with acute myocardial infarction, acute HF, right ventricular (RV) failure, left ventricular assist device (LVAD), and type 1 diabetes. We also discuss the potential mechanisms behind the cardiovascular benefits of these medications.

## 1. Introduction

Sodium-glucose co-transporter 2 (SGLT2) inhibitors are a class of medications originally used for the treatment of diabetes. SGLT2 inhibitors reduce glucose reabsorption by blocking the SGLT2 receptor in the proximal tubule, thus enhancing glycosuria and improving glycemic control [1,2,3]. SGLT2 is expressed mainly in the kidney (and to a lesser extent in the brain, liver, pancreas, and muscle) and is responsible for about 90% of glucose reabsorption, while SGLT1 is found mainly in the small intestine and accounts for the remaining 10%. The capacity/affinity for glucose transport is higher for SGLT2 than SGLT1 [3]. Recent studies have demonstrated beneficial cardiovascular and renal outcomes independent of the presence of diabetes. In the EMPA-REG outcomes (empagliflozin, cardiovascular outcomes, and mortality in type 2 diabetes) trial, empagliflozin reduced the composite outcome of cardiovascular death, nonfatal myocardial infarction, or nonfatal stroke in patients with diabetes and established cardiovascular disease [4]. In the DECLARE-TIMI 58 (dapagliflozin and cardiovascular outcomes in type 2 diabetes) trial, dapagliflozin reduced heart failure (HF) hospitalizations in patients with diabetes and established/at high risk of cardiovascular disease [5]. Positive cardiovascular effect was also observed in the CANVAS (canagliflozin and cardiovascular and renal events in type 2 diabetes) trial at the expense of increased risk of limb amputations, while the VERTIS-CV (cardiovascular outcomes with ertugliflozin in type 2 diabetes) trial failed to show that ertugliflozin has cardioprotective effects in patients with diabetes and atherosclerotic disease [6,7]. In the EMPEROR-Reduced trial, the addition of empagliflozin on top of guideline-directed medical therapy reduced HF hospitalizations [8]. In the DAPA HF (dapagliflozin in patients with heart failure and reduced ejection fraction) trial, dapagliflozin reduced the risk of HF and death from cardiovascular causes [9]. Recently, promising results were also reported in patients with mildly reduced and preserved ejection fraction [10,11]. Besides the cardiovascular benefits, SGLT2 inhibitors also had renal-protective properties, as demonstrated in the DAPA-CKD, EMPA-KIDNEY trials [12,13]. Since it is clear now that SGLT2 inhibitors’ positive effects are not related to anti-glycemic properties, more indications in special populations, such as right heart failure patients, have recently emerged. Moreover, the evidence for the benefits of early initiation observed in the major clinical trials justifies the initiation of this class of drugs as soon as possible during hospitalization. In the current review, we discuss the current evidence on the use of SGLT2 inhibitors in special populations, including patients with acute myocardial infarction (AMI), acute HF, right ventricular (RV) failure, with left ventricular assist device (LVAD), and with type 1 diabetes. Evaluating efficacy and safety in such populations will give the opportunity of improving cardiovascular outcomes in more patients. We also discuss the potential mechanisms behind the cardioprotective properties of SGLT2 inhibitors.

## 2. SGLT2 Inhibitors in Special Populations

### 2.1. Patients with Acute Myocardial Infarction

Several studies have focused on the safety and efficacy of SGLT2 inhibitors in the setting of AMI. Following AMI, patients are at increased risk of developing HF secondary to neurohormonal activation and the subsequent cardiac remodeling with altered myocardial metabolism [14]. The rationale behind the early initiation of SGLT2 inhibitors in the setting of AMI is the potential improvement in myocardial contractility, endothelial function, and cardiac tissue energy metabolism. The diuretic effect along with the modest decline in blood pressure associated with SGLT2 inhibitors lead to afterload reduction and a decrease in filling pressures. In animal models, empagliflozin reduced cardiac fibrosis when initiated early after the induction of AMI by inhibiting TGF-β1/Smad3 fibrotic pathway, probably unrelated to the hemodynamic effects of the drug [15]. In the EMMY (empagliflozin in myocardial infarction) trial, 476 patients with AMI were randomized to 10 mg empagliflozin versus placebo 72 h after percutaneous coronary intervention (PCI) [16]. Empagliflozin improved left ventricular function and diastolic parameters, and reduced N-terminal pro-brain natriuretic peptide (NT-proBNP) levels, indicating possible improved cardiovascular outcomes in the long term. Some major issues should be considered before the administration of SGLT2 inhibitors in AMI. First, patients with AMI may present with acute HF (particularly after anterior wall infarction); therefore, SGLT2 inhibitors should be initiated only after the clinical stabilization of the patient. Second, most patients are treated by PCI with the use of large volumes of contrast media, depending on the complexity and duration of the procedure. Concomitant exposure to contrast media and SGLT2 inhibitors may potentially increase the risk of contrast-induced nephropathy, especially in diabetic patients and in patients receiving diuretic medications. Third, some patients with complex coronary anatomy (including left main and three vessel disease) may be referred to cardiac surgery, and SGLT2 inhibitors should be discontinued prior to cardiac surgery to reduce the risk of ketoacidosis. Currently, two major trials are underway to assess the safety and efficacy of SGLT2 inhibitors in myocardial infarction: the EMPACT-MI (NCT04509674) and the DAPA-MI (NCT04564742). In Table 1, we summarize the current and ongoing trials targeting SGLT2 inhibitors in AMI.

### 2.2. Patients with Acute Heart Failure

The current data support the initiation of SGLT2 inhibitors in hospitalized patients with acute HF after stabilization. In the EMPA-RESPONSE-AHF trial, 80 patients with acute HF were randomized to empagliflozin or placebo within 24 h of presentation [17]. Overall, empagliflozin was safe, well-tolerated, and reduced the composite endpoint of worsening HF and rehospitalization for HF or death at 60 days. There was no effect on the dyspnea score, NT-proBNP level, or length of stay [17]. Sotagliflozin (a dual SGLT1/2 inhibitor) predominantly inhibits SGLT2 in the glomerulus and has the effect of reducing glucose absorption in the intestinal tract by inhibiting SGLT1 receptors [18]. In the SOLOIST-WHF (effect of sotagliflozin on cardiovascular events in participants with type 2 diabetes post worsening heart failure) trial, diabetic patients with eGFR > 30 mL/min/1.73 m^2^ hospitalized for acute HF with elevated natriuretic peptides were randomized for sotagliflozin versus placebo [18]. Patients were included after excluding acute coronary syndrome and only after meeting stabilization criteria for HF (on oral diuretic therapy, systolic blood pressure above 100 mmHg, and no need for intravenous inotropic or vasodilator therapy). Sotagliflozin was initiated during hospital stay or shortly after discharge (within 3 days), and resulted in lower total number of cardiovascular deaths and hospitalizations for HF, as well as an increase in the KCCQ score (Kansas City cardiomyopathy questionnaire score). In the EMPULSE trial (empagliflozin in patients hospitalized with acute heart failure who have been stabilized), 530 patients with acute HF (both diabetic and non-diabetic) were randomized to 10 mg empagliflozin versus placebo during their hospital stay [19]. Clinical benefit (defined as hierarchical endpoint of all-cause mortality, number of HF events or time to first hospitalization, or an increase in KCCQ score) was in favor of the empagliflozin group. It was shown in a recent meta-analysis of the EMPA-RESPONSE-AHF, SOLOIST-WHF, and EMPULSE trial that the early initiation of SGLT2 inhibitors reduces the risk of rehospitalization for HF and improves patient-reported outcomes with no excess risk of adverse effects [20]. The effects of early empagliflozin initiation on diuresis and kidney function in patients with acute decompensated heart failure (EMPAG-HF) trial also showed that the early addition of empagliflozin on top of standard diuretic therapy increases diuresis in patients with acute HF without jeopardizing kidney function [21]. Currently, the DAPA ACT HF-TIMI 68 [NCT04363697] and the DICTATE-AHF [22] are also evaluating the safety and efficacy of dapagliflozin in acute HF. In summary, SGLT2 inhibitors should probably be initiated early after the stabilization of acute HF, preferably during the hospital stay in order to maximize their beneficial cardiorenal effects. Table 2 summarizes the major trials focusing on SGLT2 inhibitors in acute HF.

### 2.3. Patients with Isolated Right Ventricular Failure

Currently, there is no consistent clear evidence on the role of SGLT2 inhibitors in improving RV performance. In the EMBRACE-HF trial, the use of empagliflozin was associated with reduction in pulmonary artery pressure (PAP), measured by the CardioMEMS PAP sensor regardless of loop diuretics use [23]. Furthermore, in the SIMPLE trial, empagliflozin reduced pulmonary artery capillary pressure at rest but not during exercise [24]. Consistent with these results, Kayano et al. demonstrated a decrease in RV systolic pressure during exercise in diabetic patients treated with dapagliflozin [25]. In addition, several studies have focused on the echocardiographic parameters of RV performance following the administration of SGLT2 inhibitors. Sarak et al. evaluated the effect of empagliflozin in patients with diabetes and coronary artery disease, and found that RV mass index, RV end-diastolic and end-systolic volume index, and RV ejection fraction were not changed 6 months after drug initiation of empagliflozin [26]. In another study of patients with HFREF, the addition of SGLT2 inhibitors on top of optimal medical therapy resulted in improved RV performance and a decrease in tricuspid valve regurgitation after three months of therapy [27]. The difference in the results of the studies may be attributed to possible differences in drug type characteristics, study population, and definition of endpoints. Since most studies indicate a decrease in PAP, and given the diuretic effects of SGLT2 inhibitors, it seems reasonable to use these drugs in patients with RV failure with evidence of volume overload. Further studies with hard cardiovascular endpoints are warranted.

### 2.4. Patients with LVAD

Despite optimal medical therapy, patients with advanced HF are often referred to left ventricular assist device (LVAD) implantation as a bridge to heart transplantation, bridge to recovery, or most commonly, as destination therapy [28,29]. Given the anti-remodeling properties and cardiovascular positive effects of SGLT2 inhibitors, their use in patients with LVAD as a bridge to recovery is reasonable since they can enhance cardiac recovery. In patients with LVAD as destination therapy, several issues should be taken into account when considering SGLT2 inhibitors’ use. One of the major pitfalls in LVAD patients is the magnitude of PAP and RV function. In the EMBRACE-HF trial, the use of empagliflozin was associated with reduction in PAP measured by a CardioMEMS sensor regardless of loop diuretics use [23]. These results are of paramount significance since PAP reduction may improve RV performance, alleviate volume overload, and help in reconsidering patients that have been deferred from LAVD due to elevated PAP. Cagliostro et al. reported on the adverse events experienced with the use of SGLT2 inhibitors in 18 LVAD implants [30]. Of them, three had genitourinary infections, four had driveline infections (DLI), and two patients had limb amputations with no episodes of diabetic ketoacidosis (DKA) or renal function deterioration, and with no change in weight or diuretic dose. However, as stated by the authors, DLI often affects LVAD implants and constitutes a major issue regardless of the use of SGLT2 inhibitors. Moreover, these infections predominantly originate from superficial skin sites related to bacteria such as staphylococcus aureus [31], while SGLT2 inhibitors are associated with increased risk of mainly urogenital infections secondary to enhanced glycosuria [32]. It is reasonable, given the promising good safety profile of these drugs along with their proven cardiovascular and renal outcomes, to use them more frequently in LVAD implants. Further clinical trials to evaluate the long-term outcomes are required. 

### 2.5. Type 1 Diabetes

While the efficacy and safety profile of SGLT2 inhibitors are well-established in type 2 diabetes, their use in type 1 diabetes is still a matter of debate. One of the major concerns in type 1 diabetes is the increased risk of DKA, which can be life threatening. The overall incidence of DKA related to the use of SGLT2 inhibitors is estimated to be 0.6 to 2.2 events per 10,000 person years, according to a large recent meta-analysis [33]. This potentially fatal condition has been reported in diabetic patients and may be encountered with normal blood glucose levels (euglycemic DKA, blood glucose <200 mg/dL), typically in the setting of acute infection or insulin non-compliance [34]. The pleotropic effects of SGLT2 inhibitors, particularly on the cardiovascular and renal systems, have raised the possibility of using them to help in controlling the blood glucose of patients with type 1 diabetes. In the EASE 1 trial, a low dose of empagliflozin (2.5 mg daily) as adjunct to insulin therapy resulted in increased 24 urinary glucose excretion with modest body weight and HBA_1_C reductions [35]. In the DEPICT-1 trial, both dapagliflozin 5 mg and 10 mg reduced HBA_1_C after 24 weeks of therapy in patients with type 1 diabetes [36]. A post hoc analysis of the DEPICT-1 trial also showed a reduction in UACR over 52 weeks for both doses [37]. In another study, canagliflozin reduced HBA_1_C and body weight in adults with type 1 diabetes but with increased risk of DKA [38]. Similarly, the addition of sotagliflozin to insulin contributed to diabetic control in type 1 diabetes but with increased incidence of DKA [39]. Although the above-mentioned results may indicate the renal benefits of SGLT2 inhibitors in type 1 diabetes in certain circumstances, this comes with the reduction in insulin dose, which may make patients prone to DKA. Currently, SGLT2 inhibitors as an adjunct therapy to insulin are still not approved for type 1 diabetes.

## 3. Side Effects

Overall, SGLT2 inhibitors have an excellent safety profile with few side effects, including urinary tract infections, genital fungal infections, urinary urgency, and flu-like symptoms. The most common side effect is candida genital infection that may affect about 10% of women [32]. In one population-based cohort study, the risk of both severe and non-severe urinary tract infections after the initiation of SGLT2 inhibitors was similar to other anti-glycemic medications [40]. Although there are concerns that SGLT2 inhibitors may increase the risk of bladder cancer, a clear association has not been proven and the results are inconsistent [41]. Bone fractures were also more commonly reported in patients taking canagliflozin due to decreased mineral density, although data are inconclusive [6]. Adverse events related to volume depletion were not increased in the major clinical trials compared to placebo. As we discussed before, DKA may occur among patients taking SGLT2 inhibitors due to increase in ketone bodies. DKA may be prevented by recognizing the patients with precipitating factors, including dehydration, uncontrolled diabetes, insulin pump failure, severe sepsis, and patients before major surgery. In the CANVAS trial, the risk of lower limb amputation was increased with canagliflozin use [6]. Thus, canagliflozin should be avoided in patients with known severe peripheral arterial disease with ulcers.

## 4. General Comments

Some of the limitations of the major clinical trials are the use of specific inclusion and exclusion criteria, making the conclusions not relevant to the general diabetic or heart failure population. However, the ongoing trials, including those that have been discussed in our review, focus on special populations. Another limitation of the major clinical trials is the low percentage of patients on the Sacubitril–Valsartan baseline therapy. Finally, although limited data exist on patients with Stage 4 chronic kidney disease (patients with eGFR <25 mL/min per 1.73 m^2^ were excluded), the current practice supports continuing the drug until the patient is on dialysis. Unstable patients with cardiogenic shock were also excluded from the trials.

## 5. Potential Mechanisms

**Figure 1:**
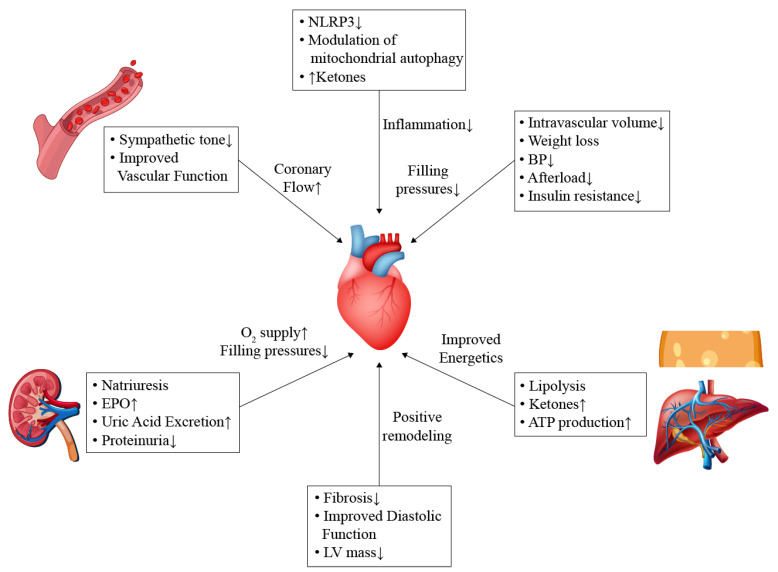
Potential mechanisms of cardiovascular benefits.

SGLT2 inhibitors exhibit pleotropic effects on the cardiovascular system. It is clear that these beneficial effects are not related to the anti-glycemic properties. Several mechanisms may explain these pleotropic properties, as illustrated in Figure 1.

Cardiovascular benefits of SGLT2 inhibitors are mediated by several pathways. Enhanced diuresis and vascular dilation result in afterload reduction and coronary blood flow augmentation. Ketogenesis mediated by adipose tissue and hepatocytes promote ketogenesis, which serves as an energy source for the failing heart. In addition, SGLT2 inhibitors contribute to decreasing inflammation and improving systolic and diastolic functions by reducing fibrosis and inhibiting remodeling pathways.

ATP, adenosine triphosphate; BP, blood pressure; EPO, erythropoietin; LV, left ventricle; NLRP3, nucleotide-binding oligomerization domain, leucine-rich repeat, and pyrin domain-containing 3.

### 5.1. General Effects

Early afterload reduction and enhanced diuresis with the concomitant decrease in filling pressures probably play a role in the rapid reduction in HF hospitalizations that have been observed in the major clinical trials. In addition, SGLT2 inhibitors also cause a modest weigh reduction that may help in reducing blood pressure and improving glycemic control. The reduction in blood pressure is suggested to be secondary to improving endothelial function and decreasing arterial stiffness and sympathetic activity (discussed below) and unrelated to volume depletion or natriuresis [42,43]. Since osmotic diuresis associated with SGLT2 inhibitors is dependent on glucose level, this effect does not explain the benefits in non-diabetic patients with HF [42]. NT-proBNP is an important prognostic marker in HF, which reflects the volume status and the overall wall stress. However, in the DEFINE-HF trial, there was no decrease in NT-proBNP concentration despite the improvement in HF status [44]. This finding raises questions about the role of natriuresis associated with SGLT2 inhibition in improving HF outcomes [42]. Likewise, weight reduction is observed mainly in diabetic patients, and therefore, does not provide an explanation for cardiovascular benefits in non-diabetic patients.

### 5.2. Effects on Sympathetic Pathways

HF is associated with the activation of several neurohormonal pathways, including the sympathetic system, renin–angiotensin–aldosterone system, and the natriuretic peptides pathways. Despite a modest decline in blood pressure and intravascular volume depletion associated with SGLT2 inhibitors, there is no concomitant increase in heart rate. This finding may indicate a possible effect on the sympathetic system [45,46]. In experimental rat models with metabolic syndrome, luseogliflozin (a selective SGLT2 inhibitor) improved the circadian rhythm of the sympathetic nervous system [47]. In another model of hypertensive mice, chemical denervation caused a reduction in blood pressure, blood glucose, and renal SGLT2 protein expression [48]. Furthermore, the inhibition of SGLT2 in these models reduced the level of tyrosine hydroxylase and norepinephrine. Evidence for improving cardiac nerve activity with empagliflozin was demonstrated in diabetic patients with AMI in the EMBODY trial [49]. These findings may give insight to the role of SGLT2 inhibitors in preventing lethal arrhythmia in the acute phase of AMI.

### 5.3. Cardio-Renal Pathways

SGLT2 inhibitors exhibit natriuretic effects by acting on SGLT and Sodium/Hydrogen exchanger 3 (NHE3) receptors in the proximal tubule, resulting in an increase in the fractional excretion of sodium [50]. Afferent arteriolar vasoconstriction caused by SGLT2 inhibitors leads to a decrease in renal blood and an increase in erythropoietin (EPO) synthesis, which in turn promotes adenosine triphosphate (ATP) production and utilization in the cardiac tissue, and may help reduce inflammation [51]. In one study, empagliflozin administration induced erythropoiesis and iron utilization mediated by increased EPO secretion [52]. Nevertheless, it should be noted that the administration of darbepoetin alfa in patients with HF leads to an increase in hematocrit but without improvement in outcomes [53]. Another potential mediator in the cardiovascular–renal arena is serum uric acid. Uric acid is elevated in patients with diabetes and considered a part of the metabolic syndrome. Hyperuricemia in diabetic patients is associated with increased risk of hypertension, cardiovascular events, and progression of diabetic kidney disease. SGLT2 inhibitors reduce uric acid concentration by enhancing its urinary excretion facilitated by glucose transporter 9 [54].

### 5.4. Modulation of Energy Sources and Inflammatory Process

Glycosuria induced by SGLT2 inhibitors combined with the decrease in insulin and the increase in glucagon levels mimic starvation and cause lipolysis and fatty acid oxygenation, leading, finally, to ketogenesis [55]. Ketone bodies levels increase in conditions with oxidative stress, such as exercise, starvation, sepsis, and HF [56]. Under physiological conditions, the metabolism of healthy hearts depends on free fatty acids and glucose, whereas in the failing heart, a shift towards increased ketone utilization occurs [57,58]. In the failing heart, ketone bodies serve as an important source of energy as they require less oxygen per molecule of the ATP generated, and may also provide protective anti-inflammatory and antioxidant properties by inhibiting the nucleotide-binding oligomerization domain, leucine-rich repeat, and pyrin domain-containing 3 (NLRP3) inflammasome [59,60,61]. The modulation of mitochondrial autophagy is another pathway that may explain SGLT2 inhibitors’ positive cardiac effects. Autophagy is a catabolic process aimed at cleaning intracellular debris through lysosomes, and it contributes to cell hemostasis; however, this pathway is impaired in diabetic patients [62]. SGLT2 inhibitors, by mimicking nutrition deprivation, can stimulate autophagy mediated by Siritin-1 and adenosine 5′ monophosphate-activated protein kinas, thus adding another cardioprotective effect [63].

### 5.5. Microvascular Function

In an insulin resistance mouse model, empagliflozin increased the coronary flow reserve, indicating a possible positive effect on vascular function [64]. In diabetic patients, treatment with empagliflozin was associated with weight loss and reduction in several cardiac biomarkers; however, there was no improvement in coronary flow velocity reserve assesses by echo Doppler [65]. In another small trial, dapagliflozin administration improved myocardial blood flow and myocardial flow reserve measured by ^13^N-ammonia PET/CT in diabetic patients with stable coronary artery disease [66]. Differences in the results may reflect different methods in assessing coronary flow or the absence of class effect.

### 5.6. Effect on Lipid Profile

By mimicking starvation conditions, the inhibition of SGLT2 induces lipolysis in the adipose tissue and causes an increase in non-esterified fatty acids, which in turn promotes cholesterol biosynthesis in the liver and finally causes the downregulation of low-density lipoprotein cholesterol (LDL-C) [67,68]. This pathway may explain the tendency towards increase in circulating LDL-C, following SGLT2 inhibitors treatment [69]. Among the different SGLT2 inhibitor drugs, canagliflozin was associated with the worst effect on the lipid profile [69]. Along with the increase in LDL-C levels, a modest increase in high-density lipoprotein (HDL-C) has been reported with the use of SGLT2 inhibitors [67,68,69]. It should be noted, however, that the cardiovascular benefits of SGLT2 inhibitors are maintained regardless of the LDL-C or HDL-C levels [70].

### 5.7. Effect on Remodeling and Fibrosis

Several echocardiographic and animal-model studies have demonstrated the anti-remodeling effects of SGLT2 inhibitors. In non-diabetic rats, empagliflozin results in reduced collagen deposition and the inhibition of a fibrosis pathway when administered early after the induction of AMI [15]. In mouse models of pressure overload (induced by transverse aortic restriction), dapagliflozin reduced fibrosis and improved cardiac systolic function [71]. The anti-fibrotic effects of SGLT2 inhibitors may be mediated by inhibiting angiotensin II pathways, which finally leads to changes in the size, shape, geometry, and function of cardiac muscles [72,73,74,75]. In the EMPA-HEART trial, empagliflozin resulted in reduced LV mass index in diabetic patients with preserved LV function and coronary artery disease [76]. Likewise, in the SUGAR-DM-HF trial, empagliflozin led to reduction in LV end-systolic and end-diastolic volumes in diabetic and prediabetic patients with HFREF [77]. In a recent meta-analysis of randomized controlled trials of cardiac remodeling, as measured by cardiac magnetic resonance imaging, SGLT2 inhibitors’ treatment showed a decrease in LV mass [78]. Global longitudinal strain measured by speckle tracking was also shown to be positively affected by SGLT2 inhibition among patients with diabetes [79]. A significant improvement in diastolic parameters was also observed in patients with HFPEF [80]. This finding may in part explain the benefit observed in the clinical trials of SGLT2 inhibitors in HFPEF [10,11]. 

## 6. Conclusions

SGLT2 inhibitors are commonly used in patients with diabetes, HF, and chronic kidney disease, owing to their positive cardiovascular and renal effects. Other than glycemic control, SGLT2 inhibitors reduce blood pressure, enhance diuresis, and cause a modest weight loss. In addition, these drugs are associated with multiple molecular and cellular effects that altogether contribute to the cardiac and reno-protective properties. As the benefits of these drugs are not directly related to glycemic control, more indications in other populations are warranted. Patients with RV failure and patients with LVAD may benefit from enhancing RV recovery, diuresis, and preserving renal function. Due to the early benefits anticipated with the use of SGLT2 inhibitors, they should be recommended early in the setting of AMI or acute HF. Current data indicate an excellent safety profile in the acute setting of HF (after stabilization) and probably after AMI. Future studies should focus on other populations, such as patients with acute pulmonary embolism, takotsubo syndrome, and myocarditis.

## Figures and Tables

**Table 1:** Major trials of SGLT2 inhibitors in myocardial infarction.

	SGLT2 Inhibitor	Population	Timing	Primary Outcomes	Results
**EMMY**	Empagliflozin	AMI with creatinine kinase > 800 iu/L	Within 3 days of PCI	Change in NTproBNP	NTproBNP reduction and improvement in echocardiographic parameters in the empagliflozin group
**EMPACT-MI**	Empagliflozin	AMI with or at high risk of HF	Within 14 days of admission	Time to first hospitalization for HF or all-cause mortality	Ongoing trial
**DAPA-MI**	Dapagliflozin	AMI (stable)	Within 7–10 days	Time to first hospitalization for HF or cardiovascular death	Ongoing trial

HF, heart failure; NTproBNP, N-terminal pro-brain natriuretic peptide; PCI, percutaneous coronary intervention.

**Table 2:** Major trials of SGLT2 inhibitors in acute HF.

	SGLT2 Inhibitor	Population	Timing	Primary Outcomes	Results
**SOLOIST-WHF**	Sotagliflozin 200–400 mg	Acute HF and diabetes	In hospital—within 3 days of discharge	Cardiovascular death and urgent visit/hospitalization for HF	Lower urgent visit/hospitalization for HF in the sotagliflozin group
**EMPA-RESPONSE**	Empagliflozin 10 mg	Acute HF	Within 24 h of presentation	Dyspnea score, diuretic response, NT-proBNP, LOS	Empagliflozin had no effect on primary outcome *
**EMPULSE**	Empagliflozin 10 mg	Acute HF	In hospital	Clinical benefit **	Clinical benefit favors empagliflozin
**EMPAG-HF**	Empagliflozin 25 mg	Acute HF	Within 12 h	Urine output	Empagliflozin increased urine output

LOS, length of stay. Patients in the whole spectrum of ejection fraction were included. * Empagliflozin reduced combined endpoint of rehospitalization for HF or death at 60 days. ** Defined as a hierarchical composite endpoint of death from any cause, number of HF events and time to first HF event, or a 5 point or greater difference in change from baseline in the KCCQ score at 90 days, as assessed using a win ratio.

## Data Availability

Not applicable.

## Abbreviations

AMI	Acute myocardial infarction
DKA	Diabetic ketoacidosis
EPO	Erythropoietin
HDL-C	High-density lipoprotein–cholesterol
HF	Heart failure
HFPEF	Heart failure with preserved ejection fraction
HFREF	Heart failure with reduced ejection fraction
KCCQ	Kansas City cardiomyopathy questionnaire
LDL-C	Low-density lipoprotein–cholesterol
LV	Left ventricle
LVAD	Left ventricular assist device
NT-proBNP	N-terminal pro-brain natriuretic peptide
PAP	Pulmonary artery pressure
PCI	Percutaneous coronary intervention
RV	Right ventricle
SGLT2	Sodium-glucose co-transporter 2
